# Rational Utilization of Sugammadex Through Quantitative Twitch Monitoring: A Process Improvement Project at a Large Academic Medical Center

**DOI:** 10.7759/cureus.85929

**Published:** 2025-06-13

**Authors:** Markus Kowalsky, Julio Montejano, Jack Pattee, Scott Rist, Ana Fernandez-Bustamante, Samuel Gilliland, Nathaen Weitzel

**Affiliations:** 1 Anesthesiology, University of Colorado School of Medicine, Aurora, USA; 2 Biostatistics and Informatics, University of Colorado School of Public Health, Aurora, USA; 3 Enterprise Analytics, University of Colorado Hospital, Aurora, USA; 4 Anesthesiology and Pain Medicine, University of California Davis School of Medicine, Sacramento, USA

**Keywords:** cost-effective practice, cost reduction, neuromuscular blockade, neuromuscular block monitoring, process improvement, quantitative neuromuscular monitoring, sugammadex

## Abstract

Background

Sugammadex rapidly became the standard of care for neuromuscular blockade reversal at our institution, leading to a substantial increase in pharmacy costs. We hypothesized that administering sugammadex in 75 mg aliquots, rather than 200 mg vials, would improve adherence to FDA dosing guidelines, reduce costs, and promote greater use of quantitative neuromuscular monitoring.

Methods

This retrospective study analyzed a departmental process improvement initiative. Baseline sugammadex use (Phase 1) was assessed between July 2021 and January 2022, during which only qualitative neuromuscular monitoring was employed. From February to August 2022 (Phase 2), pre-filled 75 mg sugammadex syringes were introduced. In Phase 3, from September 2022 to January 2023, these aliquots were used alongside quantitative twitch monitoring. The primary outcome was the average sugammadex dose per case. Patient characteristics and secondary outcomes, including neuromuscular blockade monitoring and documentation, as well as safety event reporting, were also evaluated.

Results

There were no significant differences in patient demographics or comorbidities across phases. With the introduction of 75 mg sugammadex aliquots, the average dose per case decreased by 55.4 mg (95% CI: 52.1-58.8). Following the implementation of quantitative monitoring, the dose increased by 10.8 mg (95% CI: 7.3-14.3). Documentation of neuromuscular blockade improved, and no changes in reported safety events were observed across phases.

Conclusions

The use of smaller compounded sugammadex aliquots, combined with quantitative monitoring and appropriate education, can improve neuromuscular blockade reversal practices while reducing healthcare costs.

## Introduction

Since its FDA approval in 2015, sugammadex has rapidly become an important tool in modern anesthesia practice for the reversal of neuromuscular blockade. Despite its well-documented safety profile and efficacy, its higher upfront acquisition cost compared to traditional acetylcholinesterase inhibitors, such as neostigmine, has limited its widespread adoption in certain healthcare settings [1,2].

Soon after FDA approval, our academic anesthesia department transitioned from using neostigmine/glycopyrrolate to sugammadex as the standard pharmacological reversal agent, following an internal study that demonstrated a reduced incidence of postoperative pulmonary complications [3]. In the years since, additional studies have supported the benefits of sugammadex in reducing postoperative pulmonary complications [4], postoperative nausea and vomiting [5], and in providing faster reversal of neuromuscular blockade [2]. At the same time, research has highlighted the limitations of traditional qualitative neuromuscular monitoring [6,7], prompting multiple professional societies, including the American Society of Anesthesiologists (ASA) in their 2023 guidelines, to recommend routine use of quantitative neuromuscular monitoring [8-10].

In 2021, sugammadex use was identified as a high-cost outlier within the operating room pharmacy budget at the University of Colorado Hospital. In response, a departmental task force was formed to examine and rationalize its use (Phase 1). At the time, quantitative twitch monitors were in limited supply, and sugammadex was most commonly available in 200 mg single-use vials stocked in each operating room. The task force noted that sugammadex was frequently administered in 200 mg increments, simply due to the vial size, and raised concerns about suboptimal neuromuscular blockade monitoring practices.

Following group discussions, two key opportunities for improvement were identified: (1) providing smaller sugammadex aliquots to encourage intentional dosing aligned with FDA guidelines and (2) increasing access to quantitative neuromuscular monitoring. It was anticipated that these changes would not only promote adherence to evidence-based dosing but also improve patient safety and facilitate broader adoption of quantitative monitoring, all while reducing institutional drug costs.

This report outlines our department’s phased implementation, beginning with the introduction of 75 mg sugammadex aliquots prepared in-house by pharmacy technicians (Phase 2), followed by the deployment of quantitative twitch monitors (Phase 3). We hypothesized that each phase would contribute to a reduction in drug acquisition costs, improvement in neuromuscular monitoring and reversal practices, and no significant compromise in patient safety.

## Materials and methods

The data presented in this study were collected as part of a departmental process improvement initiative, approved as secondary research by the Colorado Multiple Institutional Review Board. Support for data acquisition and statistical analysis by the departmental biostatistician was provided through a seed grant from the University of Colorado Department of Anesthesiology.

This study aimed to improve adherence to FDA-recommended sugammadex dosing guidelines and to increase the use of quantitative twitch monitoring for neuromuscular blockade assessment. Prior to implementation, sugammadex acquisition costs alone were approximately US$2.2 million annually. A multidisciplinary task force - comprising representatives from the Department of Anesthesiology, the operating room pharmacy, and hospital administration - identified that the availability of 200 mg single-use vials contributed to excess dosing and waste. Informal surveys of anesthesiology providers, including physicians, certified registered nurse anesthetists, and anesthesiologist assistants, also revealed a prevailing lack of confidence in the institution’s qualitative twitch monitoring system. This distrust was suspected to contribute to the overadministration of sugammadex.

After several task force meetings, it was determined that a combination of routine access to quantitative twitch monitoring and smaller sugammadex aliquots could promote safer, more cost-effective, and guideline-concordant dosing. These interventions were implemented sequentially across the main inpatient operating rooms at the University of Colorado Hospital. Beginning in January 2022, in-house pharmacy technicians began compounding 75 mg sugammadex syringes under sterile conditions from 500 mg vials, replacing the previously stocked 200 mg vials at each anesthetizing location. The choice of 75 mg as the aliquot dose was based on the typical patient weight distribution (50-100 kg) and the expectation that most patients would require reversal at shallow to moderate levels of neuromuscular blockade. Under these assumptions, two to three 75 mg syringes would meet FDA-recommended dosing guidelines [11].

In September 2022, following evaluation of multiple available monitoring systems, StimPod NMS540X quantitative twitch monitors (Bell Medical, Inc., St. Louis, MO, USA) were introduced in all anesthetizing locations within the main inpatient operating rooms. This device was chosen for its compatibility with simple gel electrodes in acceleromyography (AMG) and its capacity for future electromyography (EMG) use.

Phase 1 of the study spanned from July 1, 2021, to January 31, 2022, and represented baseline sugammadex utilization using 200 mg single-use vials and predominantly qualitative twitch monitoring. Phase 2, conducted from February 1 to August 31, 2022, included the introduction of 75 mg sugammadex aliquots while qualitative twitch monitors remained in use. Phase 3, from September 1, 2022, to January 31, 2023, marked the addition of quantitative twitch monitors, with continued use of the 75 mg sugammadex aliquots.

We assessed whether the study phase was associated with changes in key endpoints, including the average sugammadex dose per patient, the proportion of cases involving neuromuscular blockade, the proportion of cases in which more than one dose of rocuronium was administered, and the proportion of cases that included appropriate documentation of neuromuscular monitoring, defined as pre- and post-sugammadex values entered into the electronic medical record.

The study design approximates a natural experiment, under the assumption that patient populations across the three phases would not differ in any systematic way. To formally test this, we applied balance criteria as proposed by Zhang et al. [12], assessing whether the standardized mean difference (SMD) for each variable was less than 0.1 and whether the variance ratio for continuous variables was less than 2. All pairwise comparisons between phases (1 vs. 2, 1 vs. 3, and 2 vs. 3) were evaluated. Balance was assessed across the following variables: age, sex, BMI, ASA physical status classification, coronary artery disease, congestive heart failure, chronic renal disease, chronic obstructive pulmonary disease, cerebrovascular accident or transient ischemic attack, diabetes, hypertension, hypothyroidism, liver disease, sleep apnea, and anesthesia duration. Dysrhythmias were considered as a potential confounder but were excluded due to inconsistent documentation in the medical record.

Since no significant differences were found in baseline variables across study groups (as detailed in the Results), unadjusted models were used to evaluate associations between phase and outcome measures. The average sugammadex dose was analyzed using single linear regression, while the proportions of cases involving neuromuscular blockade, additional rocuronium administration, and appropriate monitoring documentation were each analyzed using single logistic regression. Associations between the three-phase study structure and each outcome were assessed using likelihood ratio tests. Where significant associations were observed, pairwise differences between phases were evaluated using Tukey’s post hoc correction.

## Results

A total of 29,432 patients aged over 18 who received anesthetic care during the three study phases were initially identified. Patients were excluded if they did not receive general anesthesia, both rocuronium and sugammadex, or if they did not proceed directly to the post-anesthesia care unit (PACU). After applying these criteria, 18,698 subjects remained. Of these, 536 were excluded due to missing data, resulting in a final study population of 18,162 patients. Among the remaining cases, four involved sugammadex doses exceeding 1000 mg, and two involved rocuronium doses greater than 500 mg. Each of these six records was reviewed individually, and the cases were excluded from further analysis due to either inaccurate charting or the presence of clear outliers, such as emergent reversal of neuromuscular blockade.

Table 1 presents the distribution of study covariates stratified by study phase. Table 2 and Table 3 summarize the balance diagnostics for baseline covariates, including age, sex, ASA physical status, anesthesia duration, and other patient comorbidities. As shown in Table 2, all categorical potential confounders were balanced across study phases, with SMDs below 0.1. Table 3 further demonstrates that all continuous covariates were balanced across phases, with variance ratios below 2. Given the absence of significant differences in baseline demographics or comorbidities across the three phases, we proceeded with univariable modeling to evaluate the associations between study phase and the primary endpoints, as outlined in the Methods section.

**Table 1:** Baseline demographic and clinical data for study subjects, stratified by study phase ASA, American Society of Anesthesiologists; CAD, coronary artery disease; CHF, congestive heart failure; COPD, chronic obstructive pulmonary disease; CVA, cerebrovascular accident; HTN, hypertension; TIA, transient ischemic attack

Characteristics	Phase 1 (N = 6149)	Phase 2 (N = 6970)	Phase 3 (N = 5043)	Overall (N = 18,162)
Mean age (SD, both in years)	53.7 (17.1)	53.1 (17.0)	52.7 (17.4)	53.2 (17.2)
Median age (min, max, all in years)	55.0 (18.0, 104)	55.0 (18.0, 101)	54.0 (18.0, 122)	55.0 (18.0, 122)
Female sex	3333 (54.2%)	3746 (53.7%)	2761 (54.7%)	9840 (54.2%)
Male sex	2816 (45.8%)	3224 (46.3%)	2282 (45.3%)	8322 (45.8%)
Mean BMI (SD in kg/m²)	28.6 (7.41)	28.4 (7.13)	28.5 (7.43)	28.5 (7.31)
Median BMI (min, max in kg/m²)	27.3 (12.5, 91.6)	27.2 (13.4, 81.7)	27.0 (13.7, 69.9)	27.2 (12.5, 91.6)
Mean ASA class (SD)	2.52 (0.672)	2.55 (0.663)	2.54 (0.646)	2.54 (0.661)
Median ASA class (min, max)	3.00 (1.00, 5.00)	3.00 (1.00, 5.00)	3.00 (1.00, 5.00)	3.00 (1.00, 5.00)
Asthma	1024 (16.7%)	1201 (17.2%)	879 (17.4%)	3104 (17.1%)
CAD	702 (11.4%)	714 (10.2%)	495 (9.8%)	1911 (10.5%)
CHF	328 (5.3%)	358 (5.1%)	245 (4.9%)	931 (5.1%)
Kidney disease	1217 (19.8%)	1365 (19.6%)	904 (17.9%)	3486 (19.2%)
COPD	537 (8.7%)	555 (8.0%)	356 (7.1%)	1448 (8.0%)
CVA/TIA	428 (7.0%)	484 (6.9%)	295 (5.8%)	1207 (6.6%)
Diabetes	1540 (25.0%)	1799 (25.8%)	1236 (24.5%)	4575 (25.2%)
HTN	3204 (52.1%)	3614 (51.9%)	2480 (49.2%)	9298 (51.2%)
Hypothyroidism	1173 (19.1%)	1300 (18.7%)	865 (17.2%)	3338 (18.4%)
Liver disease	622 (10.1%)	721 (10.3%)	463 (9.2%)	1806 (9.9%)
Sleep apnea	1636 (26.6%)	1793 (25.7%)	1234 (24.5%)	4663 (25.7%)
Anesthesia duration mean (SD; minutes)	208 (123)	205 (122)	198 (117)	204 (121)
Anesthesia duration median (min, max; minutes)	177 (38.0, 888)	174 (25.0, 958)	168 (28.0, 1660)	173 (25.0, 1660)
Mean rocuronium dose (mg) (SD)	94.7 (59.4)	89.4 (55.4)	86.1 (51.2)	90.3 (55.8)
Median rocuronium dose (mg) (min, max)	80.0 (0, 1040)	80.0 (0, 590)	70.0 (0, 680)	80.0 (0, 1040)
Surgical service represented				
Anesthesiology	1 (0.0%)	0 (0%)	0 (0%)	1 (0.0%)
Cardiothoracic	292 (4.7%)	326 (4.7%)	259 (5.1%)	877 (4.8%)
Dental	8 (0.1%)	12 (0.2%)	8 (0.2%)	28 (0.2%)
Gastroenterology	5 (0.1%)	8 (0.1%)	1 (0.0%)	14 (0.1%)
General	1664 (27.1%)	1812 (26.0%)	1392 (27.6%)	4868 (26.8%)
Maxillofacial/oral surgery	3 (0.0%)	1 (0.0%)	1 (0.0%)	5 (0.0%)
Neurology	1 (0.0%)	1 (0.0%)	0 (0%)	2 (0.0%)
Neurosurgery	772 (12.6%)	995 (14.3%)	629 (12.5%)	2396 (13.2%)
Obstetrics	1 (0.0%)	0 (0%)	0 (0%)	1 (0.0%)
Obstetrics and gynecology	606 (9.9%)	691 (9.9%)	499 (9.9%)	1796 (9.9%)
Ophthalmology	67 (1.1%)	71 (1.0%)	58 (1.2%)	196 (1.1%)
Orthopedics	1252 (20.4%)	1433 (20.6%)	1018 (20.2%)	3703 (20.4%)
Otolaryngology - ENT	141 (2.3%)	156 (2.2%)	119 (2.4%)	416 (2.3%)
Plastics	258 (4.2%)	294 (4.2%)	223 (4.4%)	775 (4.3%)
Podiatry	4 (0.1%)	5 (0.1%)	6 (0.1%)	15 (0.1%)
Pulmonology	18 (0.3%)	50 (0.7%)	18 (0.4%)	86 (0.5%)
Radiation oncology	11 (0.2%)	5 (0.1%)	13 (0.3%)	29 (0.2%)
Transplant	323 (5.3%)	371 (5.3%)	242 (4.8%)	936 (5.2%)
Urology	644 (10.5%)	622 (8.9%)	483 (9.6%)	1749 (9.6%)
Vascular	78 (1.3%)	117 (1.7%)	74 (1.5%)	269 (1.5%)
Mean sugammadex dose (mg) (SD)	185 (90.7)	133 (79.6)	142 (88.3)	153 (88.9)
Median sugammadex dose (mg) (min, max)	200 (0, 1200)	150 (0, 1080)	150 (0, 800)	150 (0, 1200)
Additional rocuronium dosage after the first	3531 (57.4%)	3941 (56.5%)	2889 (57.3%)	10,361 (57.0%)
Proper documentation of neuromuscular blockade	2831 (46.0%)	3400 (48.8%)	2558 (50.7%)	8789 (48.4%)

**Table 2:** SMDs for baseline covariates across all pairwise comparisons of study phases ASA, American Society of Anesthesiologists; CAD, coronary artery disease; CHF, congestive heart failure; COPD, chronic obstructive pulmonary disease; CVA, cerebrovascular accident; HTN, hypertension; SMD, standardized mean difference; TIA, transient ischemic attack

Characteristics	SMD (1 vs. 2)	SMD (1 vs. 3)	SMD (2 vs. 3)
Age (year)	0.035	0.055	0.02
Sex	0.009	0.011	0.02
BMI (kg/m²)	0.018	0.015	0.003
ASA class	0.042	0.023	0.02
Asthma	0.015	0.021	0.005
CAD	0.038	0.052	0.014
CHF	0.009	0.022	0.013
Chronic renal disease	0.005	0.048	0.042
COPD	0.028	0.062	0.034
CVA/TIA	0.001	0.045	0.045
Diabetes	0.018	0.012	0.03
HTN	0.005	0.059	0.053
Hypothyroidism	0.011	0.05	0.039
Lung disease	0.008	0.032	0.039
Apnea	0.02	0.049	0.029
Anesthesia duration	0.025	0.081	0.056

**Table 3:** Variance ratios for continuous baseline covariates across all pairwise comparisons of study phases ASA, American Society of Anesthesiologists

Characteristics	Variance ratio (1 vs. 2)	Variance ratio (1 vs. 3)	Variance ratio (2 vs. 3)
Age (years)	0.99	1.04	1.05
BMI (kg/m²)	0.93	1	1.09
ASA class	0.97	0.92	0.95
Anesthesia duration	0.98	0.91	0.93

**Figure 1:**
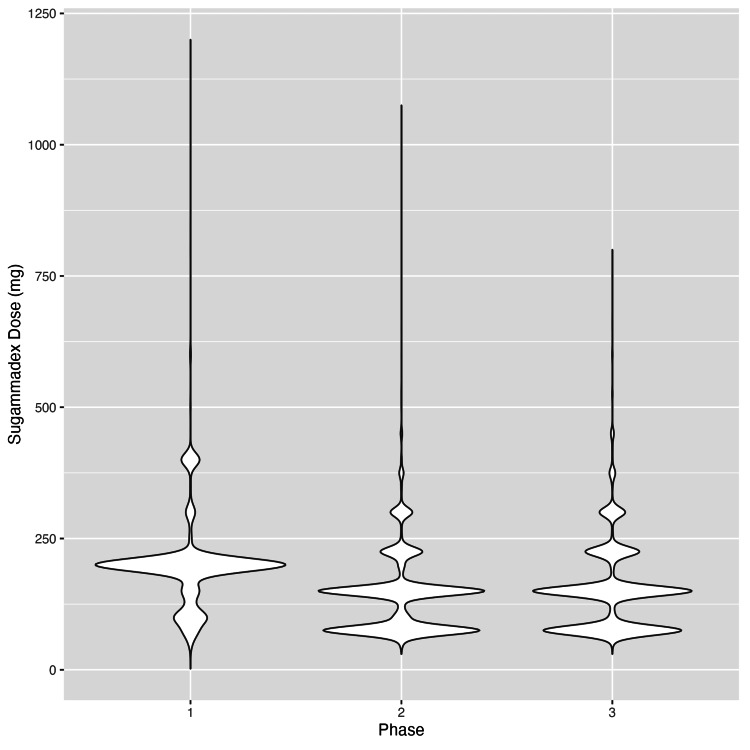
Distribution of per-patient sugammadex dosage, highlighting the most common dosing patterns stratified by study phase

The relationship between per-patient sugammadex dosage and study phase was assessed in a subset of 17,030 subjects who received a nonzero dose of sugammadex, drawn from the 18,162 subjects summarized in Table 1. A significant association was found between sugammadex dosage and study phase (p < 0.001). Following Tukey’s post hoc correction, all pairwise comparisons between phases remained statistically significant (p < 0.001). On average, patients in Phase 2 received 55.4 mg less sugammadex than those in Phase 1 (95% CI: 52.1, 58.8). Patients in Phase 3 received an average of 44.6 mg less than Phase 1 (95% CI: 41.0, 48.3), and 10.8 mg more than Phase 2 (95% CI: 7.3, 14.3). The distribution of sugammadex dosing by study phase is illustrated in Figure 1.

The relationship between additional rocuronium administration and the study phase was evaluated in 18,131 subjects who received at least one dose of rocuronium. No significant association was found between study phase and the use of an additional rocuronium dose (p = 0.53). Within this same subpopulation, we also assessed the relationship between study phase and appropriate documentation of neuromuscular blockade monitoring. A significant association was observed (p < 0.001). Following Tukey’s post hoc correction, two pairwise comparisons showed statistically significant differences: subjects in Phase 2 had 11.6% higher odds of appropriate documentation compared to those in Phase 1 (p = 0.005, 95% CI: 2.8%, 21.2%), while subjects in Phase 3 had 20.8% higher odds compared to Phase 1 (p < 0.001, 95% CI: 10.5%, 32.0%).

The relationship between the study phase and the proportion of cases utilizing neuromuscular blockade was assessed in 22,781 patients who received general anesthesia, proceeded directly to the PACU, and were not transferred to the ICU immediately following surgery. No significant association was found between study phase and neuromuscular blockade utilization (p = 0.17).

## Discussion

In this process improvement project, we describe our department’s efforts to optimize sugammadex dosing practices and promote the use of quantitative neuromuscular blockade monitoring. Our findings suggest that significant cost savings can be safely achieved through the use of pharmacy-prepared sugammadex aliquots combined with the implementation of quantitative twitch monitoring. Although acquisition costs were successfully reduced in this study, several limitations must be considered by other institutions aiming to implement a similar approach.

Technical issues (monitoring)

Although this project was initiated prior to the release of the 2023 American Society of Anesthesiologists Practice Guidelines for Monitoring and Antagonism of Neuromuscular Blockade [10], there was already a growing consensus within our department that residual neuromuscular blockade represented a clinically significant, patient-centered concern warranting further attention. In response, the department evaluated several models of quantitative twitch monitors to improve monitoring availability. Ultimately, the StimPod NMS540X (Bell Medical, Inc.) was selected for its dual capability to perform both AMG and EMG.

To control ongoing costs associated with single-use EMG electrodes, the department opted to use AMG for most cases, reserving EMG for situations in which AMG was contraindicated. Moreover, potential savings from reduced sugammadex acquisition costs were intended to offset the investment in new monitoring equipment. Within two months, the initial expense of outfitting 30 operating rooms with the new monitors was fully offset by reductions in drug costs. However, several ongoing issues have hindered widespread adoption and sustained use of the StimPod system.

As described by other clinicians, including Todd et al. [13,14], the implementation of new quantitative monitoring technology presents numerous challenges. Our experience echoed these difficulties, including problems with proper electrode placement and interpretation of monitor data, despite repeated educational initiatives via departmental meetings, quality improvement newsletters, and hands-on support from experienced clinicians. Equipment durability has also proven problematic, with frequent failures of AMG cables necessitating temporary reversion to qualitative train-of-four monitors while awaiting repairs or replacements. These setbacks have slowed the pace of adoption and undermined user confidence in the new monitoring technology.

Sugammadex aliquot preparation challenges

Before implementing a program of this nature, it is essential to consider the total costs associated with drug preparation, as these can offset the overall financial savings to the health system. For example, although our institution achieved over $984,000 in annual savings on sugammadex acquisition, part of these savings was counterbalanced by expenses related to individual supplies (e.g., syringes and needles) and pharmacy technician labor, as well as the initial costs of implementing quantitative monitoring. While overall drug waste likely decreased, the environmental impact of increased syringe usage, required to deliver doses exceeding 75 mg, should not be overlooked.

As shown in Figure 1, per-patient sugammadex dosing closely aligned with the aliquot size available. When considering the implementation of a similar system, it is important to determine the optimal dose per compounded syringe. In our case, a 75 mg aliquot was chosen by group consensus, as it was believed that multiples of this dose would better accommodate a variety of clinical scenarios compared to either the 50 mg or 100 mg options. Other authors have also advocated for smaller vial sizes to reduce cost and waste, especially in pediatric settings, where 100 mg vials could be advantageous [15]. With Merck’s patent for sugammadex set to expire in 2026, this may offer generic manufacturers an opportunity to diversify their pharmaceutical portfolios by offering smaller-dose options.

Safety monitoring

Although the cost and waste reductions were major drivers of this initiative, ensuring patient safety remained a top priority. To monitor safety outcomes, we reviewed all events reported in the hospital’s safety reporting system that mentioned sugammadex. Across all study phases, no significant trends were observed; only four clear cases of inadequate reversal were identified: one in Phase 1, two in Phase 2, and one in Phase 3. Given that the incidence of residual neuromuscular blockade is known to be higher even with quantitative monitoring, these findings likely reflect underreporting or misclassification. This underscores the importance of incorporating post-reversal neuromuscular monitoring for certain patients.

Previous studies, such as that by Edwards et al. [16], have explored the broader cost benefits of implementing quantitative neuromuscular monitoring. Their findings suggest that even without accounting for drug acquisition costs, quantitative monitoring can be a cost-effective strategy to reduce residual neuromuscular blockade.

Future directions

To further enhance the quality of neuromuscular blockade reversal and monitoring at our institution, we plan to continue refining our current strategies. Given the limitations in reliability and provider confidence associated with our predominantly AMG-based StimPod NMS540X monitors, we are actively evaluating other commercially available devices that utilize EMG. We also intend to assess the adequacy of our existing protocols by incorporating PACU monitoring of neuromuscular function. Consistent postoperative monitoring is critical due to the known variability in patient response to sugammadex, as described by Bowdle et al. [17]. We believe such monitoring will further enhance safety and support a cost-conscious approach to sugammadex administration.

Moreover, the analysis by Lin et al. [18], which modeled restrictive versus unrestrictive sugammadex use policies based on variables such as OR time and drug cost, offers valuable insights. At our institution, their findings support an unrestrictive sugammadex use policy aligned with FDA guidelines, complemented by routine post-reversal monitoring to detect recurarization or inadequate reversal. As other institutions adapt to the evolving landscape of neuromuscular blockade monitoring and prepare for changes in sugammadex pricing following the expiration of its patent in 2026, it will be critical to weigh each of these factors carefully.

## Conclusions

This study highlights our institution’s experience in evolving our approach to neuromuscular blockade monitoring and reversal with sugammadex. The initiative was implemented safely and cost-effectively across our large academic medical center, facilitated by pharmacy-compounded sugammadex aliquots and the integration of quantitative twitch monitoring. Since implementation, national and international anesthesiology societies have issued updated guidelines, and additional research has emerged that reinforces the importance of ongoing evaluation and adaptation of our practices. Our experience also suggests that similar process improvement projects could be extended to other high-cost or frequently wasted medications, promoting rational use and enabling the adoption of broader quality improvement initiatives.
